# Ferulic Acid Interferes with Radioactive Intestinal Injury Through the DJ-1-Nrf2 and Sirt1-NF-κB-NLRP3 Pathways

**DOI:** 10.3390/molecules29215072

**Published:** 2024-10-26

**Authors:** Xuemei Zhang, Haoyu Zhang, Mingyue Huang, Yu Mei, Changkun Hu, Congshu Huang, Huiting Zhang, Xue Wei, Yue Gao, Zengchun Ma

**Affiliations:** 1 Department of Pharmaceutical Sciences, Beijing Institute of Radiation Medicine, Beijing 100850, China; zxm190306@163.com (X.Z.); zhy16645122363@163.com (H.Z.); 15907489303@163.com (M.H.); mymeiyv@163.com (Y.M.); changkunhu@126.com (C.H.); huangcongshu0513@163.com (C.H.); viciry00@163.com (H.Z.); 17861175821@163.com (X.W.); 2 School of Traditional Chinese Medicine, Guangdong Pharmaceutical University, Guangzhou 510720, China

**Keywords:** radiation injury, intestine, ferulic acid, oxidative damage, inflammatory injury

## Abstract

Radiation-induced intestinal injury is a common complication of radiotherapy for abdominal and pelvic malignancies. Due to its rapid proliferation, the small intestine is particularly sensitive to radiation, making it a critical factor limiting treatment. Ferulic acid (FA), a derivative of cinnamic acid, exhibits antioxidant, anti-inflammatory, and anti-radiation properties. In this study, we established a mouse model of radiation-induced intestinal injury using a dose of 11 Gy at a rate of 96.62 cGy/min. Our findings indicate that FA’s protective effects against radiation-induced intestinal injury may be mediated through the parkinsonism-associated deglycase (DJ-1) nuclear factor erythroid 2-related factor 2 (Nrf2) and silent mating type information regulation 2 homolog 1 (Sirt1) nuclear Factor kappa-light-chain-enhancer of activated B cells (NF-κB) NOD-like receptor family, pyrin domain containing 3 (NLRP3). FA was found to mitigate changes in oxidative stress indices and inflammatory factors induced by radiation, as well as to attenuate radiation-induced pathological alterations in the small intestine. Furthermore, FA enhanced the expression of DJ-1 and Nrf2 at both the transcriptional and protein levels, inhibited NLRP3 protein fluorescence intensity, and reduced the expression of NLRP3, interleukin-18 (IL-18), and interleukin-1 beta (IL-1β). Additionally, FA suppressed the transcription and translation of NF-κB, NLRP3, cysteine-aspartic acid protease-1 (Caspase-1), IL-18, and IL-1β by upregulating Sirt1, thereby alleviating radiation-induced inflammatory injury in the small intestine. Thus, FA holds promise as an effective therapeutic agent for ameliorating radiation-induced intestinal injury.

## 1. Introduction

Radiation therapy plays a crucial role in the treatment of abdominal and pelvic malignancies. Despite significant efforts to minimize the adverse effects of ionizing radiation, damage to surrounding normal tissues remains inevitable [1]. The small intestine, characterized by its rapid cellular proliferation, exhibits heightened sensitivity to radiation, which can directly damage intestinal cells, leading to a cascade of consequences. Specifically, radiation exposure may result in the apoptosis of intestinal epithelial cells and intestinal crypt stem cells. Additionally, it can compromise the integrity of the epithelial barrier, resulting in digestion and absorption disorders as well as electrolyte imbalances [2]. Given the largely unclear underlying mechanisms of radioactive intestinal injury, no clinically effective drugs are currently available. Parkinson disease (autosomal recessive, early onset) 7, also known as PARK7 or DJ-1, was initially identified in association with a form of Parkinson’s disease [3]. Since then, DJ-1 has been demonstrated to play a critical role in oxidative stress response, anti-apoptotic signal transduction, and transcriptional regulation as a ubiquitous cellular protective protein [4,5]. Nrf2 is a primary regulator of the antioxidant gene response, and DJ-1 is involved in its stabilization by modulating its interaction with the repressor kelch-like ech-associated protein 1 (Keap1) and the subsequent ubiquitination [6]. Additionally, the relevant literature indicates that DJ-1 is crucial for maintaining the homeostasis of the epithelial barrier in colitis [4], and can alleviate intestinal mucosal injury caused by myocardial ischemia-reperfusion through regulating the Keap1/Nrf2 pathway in rats [7].

Sirt1, a type of nicotinamide adenine dinucleotide (NAD+)-dependent deacetylase, can regulate inflammation by interacting with the RelA (p65) subunit of NF-κB to deacetylate histones. It can also inhibit inflammatory factors by deacetylating P65 at Lysine 310 (Lys310), resulting in the transcriptional repression of various inflammation-related genes [8,9]. This interaction mitigates the inflammatory damage caused by radiation in mesenchymal stem cells by suppressing the NLRP3 inflammasome [10], which is a multi-protein complex. The NLRP3 inflammasome recognizes apoptosis-associated speck-like proteins containing CARD (ASC) and recruits caspase-1 to promote the release of downstream inflammatory factors IL-1β and IL-18, which, in turn, trigger a series of inflammatory responses [11]. Recent research has reported that the activation of the NLRP3 inflammasome plays a role in the occurrence and development of radiation-induced intestinal injury [12].

FA is a naturally occurring plant phenolic compound widely found in vegetables and fruits, and it is also the main active component in many traditional Chinese medicinal materials [13]. Our previous work demonstrated that FA is the primary active ingredient of Si-Wu-Tang. In previous studies, we have shown that FA alleviates radiation-induced immune damage via the JAK/STAT signaling pathway [14]; it suppresses apoptosis and oxidative stress via the ERK pathway, thereby diminishing radiation-induced oxidative stress in lymphocytes [15]. FA also exerts neuroprotective effects against radiation-induced neural damage by inhibiting the transcription and expression of NLRP3 in hippocampal tissues and microglia, thus reducing the secretion of pro-inflammatory cytokines [16]. Other research groups have also studied FA, demonstrating its capacity to reduce neuronal apoptosis and inflammatory cell infiltration, thereby promoting the repair of damaged sciatic nerves by inhibiting the TLR4/NF-κB signaling pathway [17]. FA is widely recognized for its antioxidative, anti-inflammatory [18], and anti-radiation effects, and it has also been proven to play significant roles in an anti-diabetes, anti-cancer, and anti-aging agent [19]. Compared to other natural products with anti-inflammatory and antioxidative properties (such as curcumin, resveratrol, and anthocyanins), FA exhibits a broader spectrum of effects, better bioavailability, and is widely available and easily ingestible. Therefore, it is necessary to further study the role of FA in radiation-induced intestinal injury. In this study, we focus on whether FA exerts protective effects against radiation-induced intestinal injury through the DJ-1-Nrf2 and Sirt1-NF-κB-NLRP3 pathways.

## 2. Results

### 2.1. Effects of FA on MDA and SOD Content in Mice

**Figure 1:**
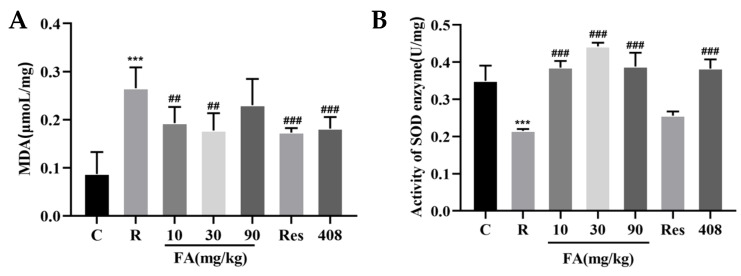
Effect of FA on oxidative stress index of intestines after radiation. (**A**) Effect of FA on MDA content in the intestines of irradiated mice. (**B**) Effect of FA on SOD content in intestines of irradiated mice. Data are expressed as mean ± SD (*n* = 3). Compared with control group, *** *p* < 0.001; compared with the radiation group, ## *p* < 0.05 and ### *p* < 0.001 (C: control; R: radiation; FA: ferulic acid; Res: resveratrol).

MDA serves as a marker of lipid peroxidation induced by oxidative stress. Seven days post-radiation, the MDA content in the radiation group increased significantly compared to the control group (Figure 1A, *p* < 0.01). The MDA levels in the FA (10 mg/kg and 30 mg/kg), Res, and 408 groups were markedly lower than those in the Radiation group (Figure 1A, *p* < 0.01). SOD is an endogenous antioxidant that inhibits oxidation processes and plays a crucial role in mitigating oxidative stress damage. Our analysis revealed that radiation significantly decreased SOD content in small intestine tissue (Figure 1B, *p* < 0.01). Conversely, the FA and 408 treatments significantly increased SOD levels (Figure 1B, *p* < 0.01).

### 2.2. Effects of FA on TNF-α and IL-6 Content in Mice

**Figure 2:**
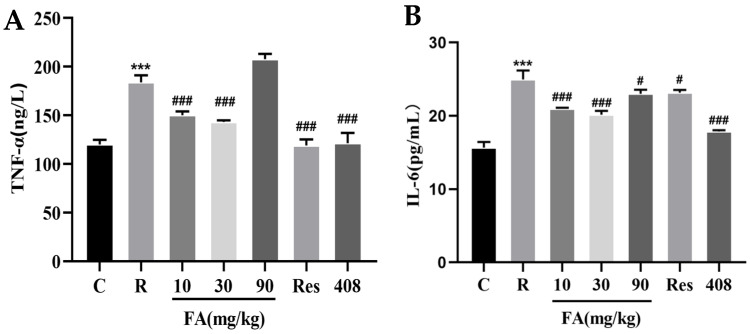
Effect of FA on the expression of inflammatory factors in the intestines of mice after radiation. (**A**) Effect of FA on TNF-α content in the intestines of irradiated mice. (**B**) Effect of FA on IL-6 content in the intestines of irradiated mice. Data are expressed as mean ± SD (n = 3). Compared with control group, *** *p* < 0.001; compared with the radiation group, # *p* < 0.05 and ### *p* < 0.001.

In the radiation group, the content of TNF-α increased dramatically compared to the control group (Figure 2A, *p* < 0.01). The administration of FA (10 mg/kg and 30 mg/kg), Res, and 408 significantly suppressed the radiation-induced elevation of TNF-α levels (Figure 2A, *p* < 0.01). Additionally, an elevated level of IL-6 was observed in the radiation group (Figure 2B, *p* < 0.01). However, this increase in IL-6 was significantly inhibited by the FA, Res, and 408 treatments (Figure 2B, *p* < 0.05 or *p* < 0.01).

### 2.3. Effects of FA on Post-Radiation Intestine Tissue Injury in Mice

**Figure 3:**
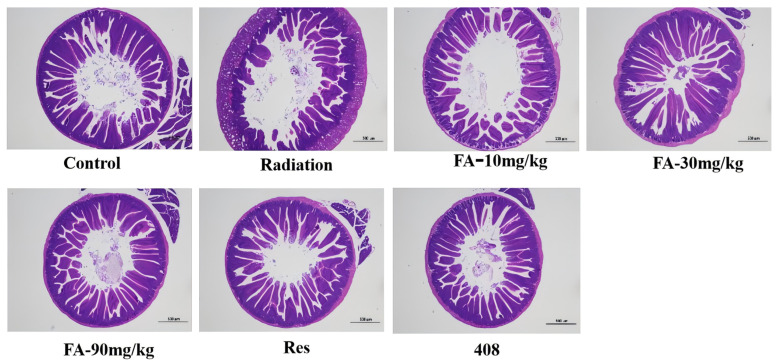
H&E stained images showing the intestinal morphology in cross-sections of the intestine. Comparisons of histopathological characteristics of hippocampal tissue among the seven group. Scale bar = 500 μm.

The control group exhibited normal intestinal tissue with intact villi and crypt structures, as observed through HE staining. In contrast, the radiation group demonstrated significant damage to the crypt structures of the intestinal mucosa, with broken and shortened villi and a loss of crypt structure. Treatment with FA, Res, and 408 resulted in the reduced fragmentation of the small intestinal villi and deepened crypt structures. These findings suggest that FA may alleviate radiation-induced intestinal tissue damage (Figure 3).

### 2.4. Effects of FA on mRNA and Protein Expression Levels of DJ-1 and Nrf2 in Post-Radiation Intestine Tissue in Mice

**Figure 4:**
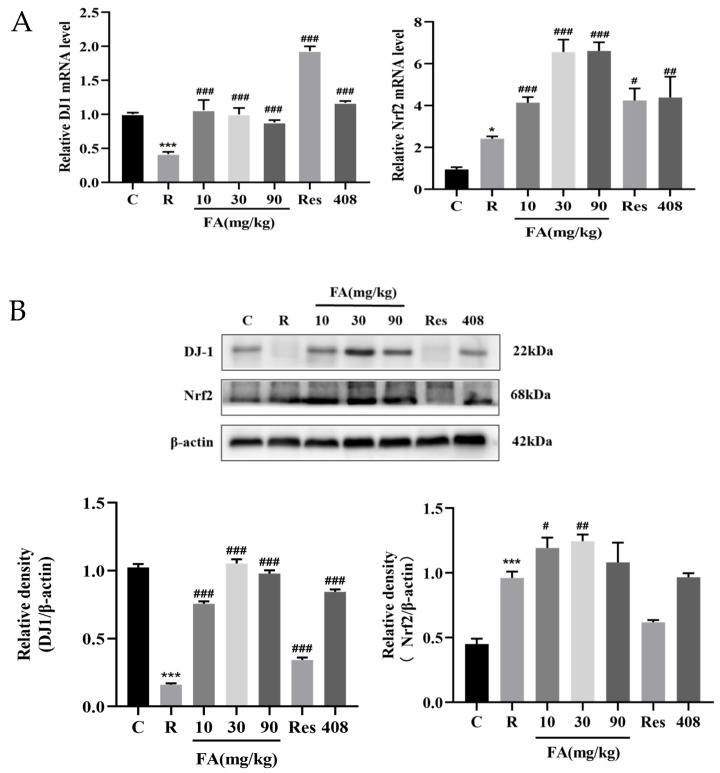
FA activates the DJ-1-Nrf2 pathway in the intestines of mice after radiation. (**A**) The mRNA level of DJ-1 and Nrf2 were detected by qRT-PCR. Data are expressed as mean ± SD (n = 3). (**B**) The protein expression of DJ-1 and Nrf2 were detected by Western blot assay. Compared with control group, *** *p* < 0.001, * *p* < 0.05; compared with radiation group, # *p* < 0.05, ## *p* < 0.05, and ### *p* < 0.05.

Radiation-induced intestinal injury is associated with oxidative stress. To explore the effects of FA on the oxidative stress pathway DJ-1-Nrf2 in relation to intestinal injury following radiation, Q-PCR and Western blot assays were conducted. FA significantly increased the mRNA levels of DJ-1 and Nrf2 in the intestine post-radiation (Figure 4A, *p* < 0.01). Moreover, FA enhanced the protein expression levels of DJ-1 and Nrf2 in the intestine following radiation (Figure 4B, *p* < 0.05 or *p* < 0.01). These findings suggest that the mechanism by which FA mitigates radiation-induced intestinal injury may involve the DJ-1-Nrf2 pathway.

### 2.5. FA Inhibited the Expression of NLRP3 Protein in Post-Radiation Intestine Tissue in Mice

**Figure 5:**
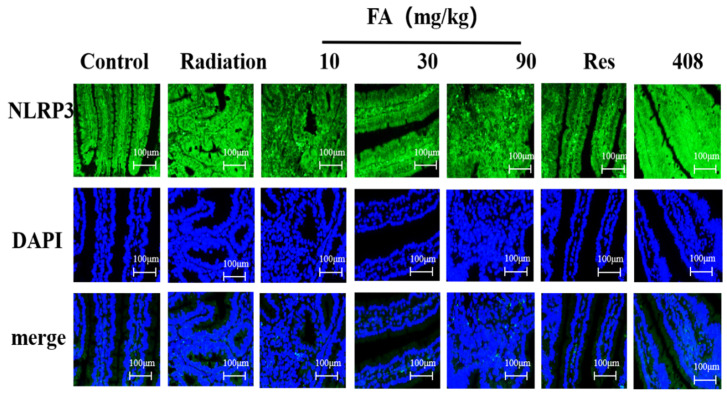
FA inhibited the expression of NLRP3 protein in the intestines of radiation mice by immunofluorescence assay. The expression and localization of NLRP3 in intestine were analyzed by immunofluorescence. All magnifications: ×400.

To confirm the role of FA on the NLRP3 inflammasome activation in the intestines of mice following radiation, we performed an immunofluorescence assay targeting the NLRP3 protein. The results demonstrated that green fluorescence expression in the radiation group was significantly increased compared to the control group (Figure 5), suggesting an upregulation of the NLRP3 inflammasome in the small intestine post-radiation. Conversely, pretreatment with FA resulted in a reduction of green fluorescence compared to the radiation group, indicating that FA can inhibit the activation of the NLRP3 inflammasome to some extent after radiation exposure.

### 2.6. Effects of FA on the Levels of NLRP3, IL-18 and IL-1β in Intestine in Mice

**Figure 6:**
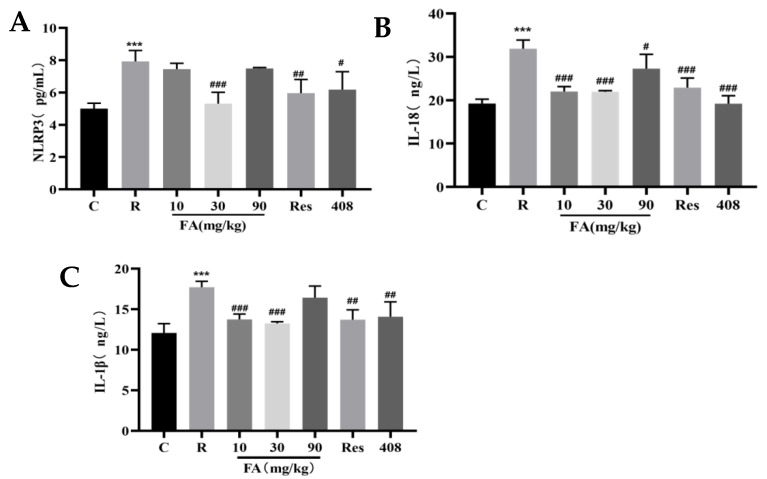
Effects of FA on related protein in the intestines of mice after radiation. (**A**) Effect of FA on NLRP3 protein expression after radiation. (**B**) Effect of FA on IL-18 protein expression after radiation. (**C**) Effect of FA on IL-1β protein expression after radiation. Compared with control group, *** *p* < 0.001; compared with radiation group, # *p* < 0.05, ## *p* < 0.05, and ### *p* < 0.05.

ELISA was employed to further verify whether FA inhibited NLRP3 inflammasome activation. The protein expression levels of NLRP3, IL-18, and IL-1β in the intestinal tissues of mice post-radiation were significantly elevated compared to the control group (Figure 6, *p* < 0.001). This elevation was reversed following pretreatment with 30 mg/kg FA (Figure 6, *p* < 0.001).

### 2.7. Effects of FA on mRNA and Protein Expression Levels of Sirt1-NF-κB-NLRP3 Pathways in Post-Radiation Intestine Tissue in Mice

**Figure 7:**
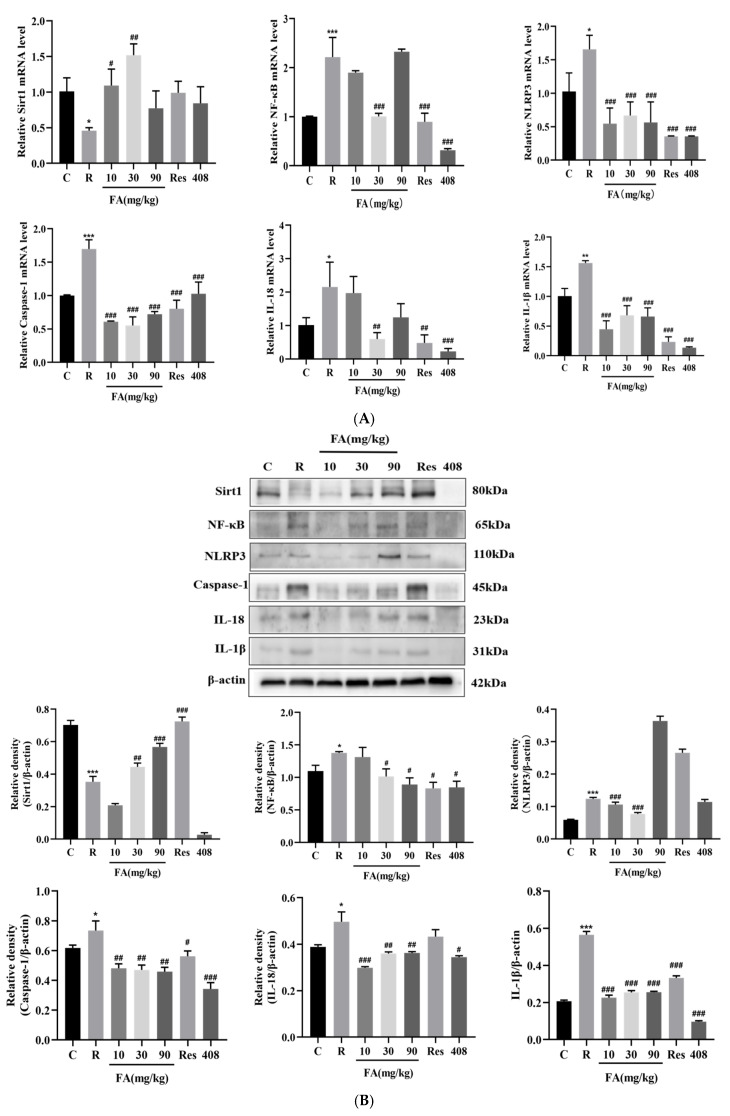
FA activates the Sirt1-NF-κB-NLRP3 pathway in the intestines of mice after radiation. (**A**) The mRNA level of Sirt1, NF-κB, NLRP3, Caspase-1, and IL-18, IL-1β were detected by qRT-PCR. Data are expressed as mean ± SD (n = 3). (**B**) The protein expression of Sirt1, NF-κB, NLRP3, Caspase-1, and IL-18, IL-1β were detected by Western blot assay. Compared with control group, * *p* < 0.05, ** *p* < 0.1, and *** *p* < 0.001; compared with radiation group, # *p* < 0.05, ## *p* < 0.01, and ### *p* < 0.001.

Radiation-induced intestinal injury is associated with inflammatory responses. The effects of FA on the inflammatory injury pathway involving Sirt1-NF-κB-NLRP3, which is related to radiation-induced small intestinal injury, were investigated using Q-PCR and Western blot analyses. FA significantly increased the mRNA levels of Sirt1 in the small intestine post-radiation and reduced the mRNA levels of NF-κB, NLRP3, Caspase-1, IL-1β, and IL-18 (Figure 7A, *p* < 0.05 or *p* < 0.01). Consistently, upon FA administration, the protein expression levels of Sirt1 in the small intestine post-radiation were significantly elevated, while the levels of NF-κB, NLRP3, Caspase-1, IL-1β, and IL-18 were markedly reduced (Figure 7B, *p* < 0.05 or *p* < 0.01). These findings suggest that the mechanism by which FA alleviates radiation-induced intestinal injury may involve the modulation of the Sirt1-NF-κB-NLRP3 pathway.

## 3. Discussion

The results of this study underscore the significant impact of ionizing radiation on the small intestine [20], one of the most rapidly renewing tissues in mammals [1]. Our findings reveal that radiation therapy targeting abdominal and pelvic malignancies substantially disrupts intestinal homeostasis, resulting in a series of adverse effects. Crucially, our research elucidates the role of ferulic acid (FA), a cinnamic acid derivative, in mitigating these effects. The extended presence of FA in the bloodstream, coupled with its low toxicity, enables it to perform protective physiological functions, such as anti-inflammatory and antioxidant activities [21]. These properties highlight its potential therapeutic application in ameliorating radiation-induced intestinal damage, a conclusion that aligns with FA’s established applications across various industries [21]. This study provides a detailed analysis of FA’s ability to maintain intestinal barrier integrity and bolster immune response under the stress conditions induced by radiotherapy. In this study, mice were locally irradiated with 60Co γ rays at a dose of 11 Gy to induce tissue oxidation and inflammatory damage. After radiation exposure, the body produces reactive oxygen species (ROS); however, when the generation of ROS exceeds the body’s capacity to neutralize them, oxidative stress ensues [22]. MDA is a well-established marker of lipid peroxidation. Concurrently, antioxidant enzymes such as SOD play a crucial role in scavenging reactive ROS from tissues. The activity levels of MDA and SOD in the small intestines of irradiated mice were significantly altered post-irradiation, but these changes were markedly reversed following the administration of FA. This suggests that FA mitigates oxidative damage in the small intestine by enhancing the antioxidant profiles of irradiated mice. Previous studies have demonstrated that the radiation-induced overproduction of ROS activates inflammasomes, leading to inflammation. The resultant imbalance in inflammatory cytokines exacerbates radiation-induced damage [23,24]. TNF-α and the IL-6 were detected, exhibiting significant increases in the small intestines of irradiated mice. However, these levels were markedly reduced following FA administration. Collectively, these findings indicate that FA can alleviate radiation-induced intestinal inflammation by reducing the production of inflammatory cytokines. H&E staining revealed that FA markedly improved the intestinal crypt structure and increased the height of the small intestinal villi.

To investigate the specific pathways involved in radiation-induced intestinal tissue oxidation and inflammatory damage, we examined the underlying mechanisms. Nrf2 is a crucial regulator of cellular redox homeostasis. Under pathological conditions, Nrf2 can trigger the transcriptional activation of downstream antioxidant genes through nuclear translocation [25]. Activation of Nrf2 has been reported to mitigate radiation-induced intestinal injury [26]. FA can respond to radiation-induced ROS-mediated oxidative stress and DNA damage by facilitating the nuclear translocation of Nrf2 and initiating the non-homologous end joining (NHEJ) repair pathway [27]. DJ-1, a multifunctional protein, is critical for regulating Nrf2 biological activity, which consequently affects antioxidant enzymes [28,29]. DJ-1 deficiency can exacerbate inflammatory bowel disease, as evidenced by increased intestinal inflammation and heightened apoptosis of intestinal epithelial cells [4,30]. Conversely, the upregulation of the DJ-1/Nrf2 pathway can ameliorate intestinal mucosal barrier dysfunction caused by myocardial ischemia-reperfusion injury [7]. Protocatechuic acid protects gastrointestinal mucosal cells from ketoprofen-induced oxidative stress through the overexpression of DJ-1/Nrf2, while si-DJ-1 blocks the increase in antioxidant enzyme expression [31]. In this study, FA was found to significantly alleviate radiation-induced oxidative damage in small intestinal tissue by upregulating DJ-1 and Nrf2 protein expression, suggesting that the DJ-1-Nrf2 pathway could play a pivotal role.

NLRP3 binds to ASC and caspase-1 to assemble the NLRP3 inflammasome, which promotes the maturation of caspase-1 [32,33]. The maturation of caspase-1 ultimately facilitates the processing and production of pro-inflammatory cytokines IL-1β and IL-18, leading to inflammatory organ damage. In the gastrointestinal tract, NLRP3 is ubiquitously expressed and is essential for maintaining intestinal homeostasis [34]. However, the overexpression of the NLRP3 inflammasome often results in severe inflammatory diseases, such as inflammatory bowel diseases (Crohn’s disease and ulcerative colitis) or intestinal invasive bacterial infections. Inhibition of NLRP3 inflammasome activation has been shown to partially protect against inflammatory damage [35,36,37]. Based on this, we performed immunofluorescence experiments and observed a dramatic increase in NLRP3 protein expression in the small intestine following irradiation. After administration of FA, the fluorescence intensity of the NLRP3 protein was significantly decreased, suggesting that FA could ameliorate radiation-induced intestinal inflammation by inhibiting NLRP3 inflammasome activation. To confirm this hypothesis, the levels of NLRP3, IL-18, and IL-1β in the small intestine were measured using ELISA.

Notably, the expression levels of NLRP3, IL-18, and IL-1β increased following radiation exposure and decreased significantly after the administration of FA, suggesting that radiation activates the NLRP3 inflammasome in the small intestine and promotes the release of downstream IL-18 and IL-1β. FA, in turn, appears to ameliorate the radiation-induced inflammatory response in the small intestine by inhibiting this process. NF-κB is activated by acetylation and phosphorylation, initiating transcriptional cascades of various target genes that play a vital role in mediating numerous cellular signal transductions involved in immune and inflammatory responses. Activation of Sirt1 results in the deacetylation of NF-κB, which inhibits NLRP3 inflammasome activation and pro-inflammatory cytokine production [38]. FA has been shown to improve spermatogenesis disorders by reversing Sirt1 expression, maintaining testosterone levels, reducing oxidative stress, and regulating PARP1 and cytoplasmic calcium concentrations [39]. Furthermore, the regulation of phosphorylation and the activation of the iκB kinase alpha/beta (IKKα/β) and inhibitor of kappa B alpha (IκBα) pathways, along with downstream NF-κB nuclear translocation, improves radiation-induced inflammation by inhibiting the expression of COX-2 and inducible nitric oxide synthase 2 (iNOS-2) [32]. Therefore, we hypothesized that Sirt1 inhibits the activation of the NLRP3 inflammasome through the deacetylation of NF-κB, a pathway that plays a crucial role in radiation-induced intestinal inflammatory damage. To test this hypothesis, Q-PCR and Western blot experiments were performed. The results indicated that FA significantly increased Sirt1 mRNA levels and decreased radiation-induced protein expression levels, reversing the changes in the mRNA and protein expression levels of NLRP3, caspase-1, IL-18, and IL-1β.

In conclusion, FA ameliorates radiation-induced oxidative and inflammatory damage in intestinal tissue through the overexpression of the DJ-1-Nrf2 pathway and the inhibition of NLRP3 inflammasome activation. This process reduces the production of pro-inflammatory factors IL-18 and IL-1β, thereby mitigating radiation-induced oxidative and inflammatory damage in intestinal tissue.

## 4. Materials and Methods

### 4.1. Reagents

Ferulic acid (No. 90034) with a purity of 99.0% was obtained from Sigma-Aldrich (St. Louis, MO, USA). Resveratrol (No. 501-36-0) with a purity of 98.0% was collected from Shanghai Yuanye Biological Technology Co., Ltd. (Shanghai, China).

### 4.2. Mice and Treatment

Male C57BL/6J mice (20 ± 2 g; 6–8 weeks old) were purchased from Beijing Vital River Laboratory Animal Technology Co., Ltd. (Beijing, China; Certificate No. SCXK 2016-0006). All mice were housed under 12 h/12 h light/dark cycle conditions at a constant temperature and were provided free access to standard food and water. The mice were randomly divided into seven groups, each consisting of twelve mice. The drug was administered via oral gavage at a dosage of 0.1 mL/10 g body weight for seven days following one week of adaptive feeding. After four days of preventive administration, and one hour after the final dose, the mice were irradiated and subsequently given the drug via oral gavage for three consecutive days. The experimental groups were as follows: control (distilled water), radiation (distilled water), radiation + resveratrol (30 mg/kg), radiation + 408 (100 mg/kg), radiation + FA (10 mg/kg), radiation + FA (30 mg/kg), and radiation + FA (90 mg/kg).

### 4.3. Radiation

The mice were weighed before anesthesia and anesthetized with 0.4% pentobarbital sodium (0.3 mL/20 g body weight) via intraperitoneal injection. During the experiment, the abdominal area of each mouse was exposed to an 11 Gy dose of ^60^Co γ-rays at a rate of 96.62 cGy/min, with the rest of the body shielded by a lead board. This irradiation was performed using a ^60^Co γ-ray source from the Beijing Institute of Radiology Medicine, China. The control group underwent the same procedures except for the radiation exposure.

### 4.4. Sample and Tissue Preparation

The mice were euthanized 7 days post-irradiation. Small intestine tissue samples were collected and fixed in 4% formalin for hematoxylin and eosin (HE) staining. The remaining small intestine tissue samples were stored in cryopreservation tubes at −80 °C for subsequent analysis.

### 4.5. Measurement of MDA and SOD Content

The malondialdehyde (MDA) content was measured using a lipid oxidation detection kit. The principle underlying this assay involves a colorimetric response, where MDA reacts with thiobarbituric acid (TBA) to produce a red chromogen. Subsequently, the MDA content in the small intestine tissue samples was quantified using a colorimetric method at 553 nm. The superoxide dismutase (SOD) content was assessed using a total SOD activity detection kit (WST-8 method). This method is based on a colorimetric reaction involving WST-8, which reacts with superoxide anions (O^2−^) in the presence of xanthine oxidase (XO) to form a water-soluble formazan dye. The colorimetric analysis of the WST-8 product allows for the determination of SOD activity, as SOD inhibits this reaction.

### 4.6. Intestine Tissue Inflammatory Cytokines TNF-α and IL-6 Detection

A suitable amount of small intestine tissue was homogenized at 25 Hz for 5 min using a tissue homogenizer. Subsequently, the supernatant was collected by centrifugation at 12,000 rpm for 15 min. The levels of inflammatory cytokines in the intestinal tissue supernatant were then measured using ELISA kits for mouse tumor necrosis factor α (TNF-α) (Catalog No. MM-0132M1, MEIMIAN, Yancheng, China) and interleukin-6 (IL-6) (Catalog No. MM-0163M1, MEIMIAN, Yancheng, China).

### 4.7. Histological Analysis

Seven days post-irradiation, the small intestine tissue samples were fixed in 4% paraformaldehyde solution for 24 h. Following dehydration in a graded series of ethanol concentrations, the tissues were rolled into “Swiss rolls” and sectioned at 3 μm after paraffin embedding. The paraffin sections were then deparaffinized, rehydrated, and washed with double-distilled water. Histological analysis was conducted using H&E staining according to the manufacturer’s instructions. Morphological changes in the small intestine were observed using a Nikon Eclipse C1 microscope (Nikon, Shanghai, China).

### 4.8. Immunofluorescence Detection of NLRP3 Protein Expression

After the paraffin sections of the small intestine were deparaffinized and rehydrated, the sections were subjected to antigen retrieval by boiling in 0.1 M citrate buffer (pH 6.0). Subsequently, a self-fluorescence quenching agent was applied for 5 min. The sections were then blocked with 5%thht BSA (No. GC305010, Servicebio, Wuhan, China) for 30 min at room temperature and incubated with NLRP3 antibody (No. 19771-1-AP, Proteintech, Chicago, IL, USA) overnight at 4 °C. Following three washes with PBS, the sections were incubated with a secondary antibody for 50 min at room temperature, protected from light. After an additional three washes with PBS, DAPI staining solution was applied to the sections for 10 min in the dark at room temperature. The sections were then washed with PBS three times, and an anti-fade mounting medium was applied. Finally, the sections were observed under a fluorescence microscope, and images were captured.

### 4.9. The Expression Levels of NLRP3, IL-18 and IL1β Were Detected by ELISA

The appropriate amount of small intestine tissue was collected and homogenized at 25 Hz for 5 min to ensure thorough grinding. The homogenate was then centrifuged at 12,000 rpm for 15 min, and the supernatant was collected. To quantify the levels of inflammatory cytokines, the intestinal tissue supernatant was analyzed using Enzyme-Linked Immunosorbent Assay (ELISA) kits specifically designed for NLRP3 (No. MM-0656M1, MEIMIAN, Yancheng, China), IL-18 (No. MM-0169M1, MEIMIAN, Yancheng, China), and IL-1β (No. MM-0040M1, MEIMIAN, Yancheng, China).

### 4.10. Quantitative Real-Time PCR

Total RNA was extracted using TRIzol reagent (No. AG21101, Accurate, Shanghai, China) and reverse transcribed with the One-Step gDNA Removal and cDNA Synthesis SuperMix Kit (No. AT3311-02, TransGen Biotech, Beijing, China) following the manufacturer’s instructions. A real-time polymerase chain reaction (PCR) was conducted using a Green quantitative polymerase chain reaction (qPCR) SuperMix Kit (No. AQ101-03, TransGen Biotech, Beijing, China) on a StepOne™ Real-Time PCR detection system (No. 4376600, Applied Biosystems, Foster City, CA, USA). The relative expression levels of the target genes were normalized to β-actin as an internal control. The primers used in this study were synthesized and are listed in Table 1.

### 4.11. Western Blot

Proteins from intestinal tissue in different groups were extracted using lysis buffer (No. C1053+-100, Applygen, Beijing, China) containing protease and phosphatase inhibitors. The protein concentrations were then quantified using the Bicinchoninic Acid (BCA) assay (No. PC0020, Solarbio, Beijing, China), and the proteins were diluted to uniform concentrations. Protein levels were detected by Western blotting. Briefly, after electrophoresis on 10% SDS-PAGE gels, the proteins were transferred to a PVDF membrane (No. R1MB58396, Millipore, Bedford, MA, USA). Subsequently, the membranes were blocked with 5% non-fat milk for 3 h at room temperature and incubated overnight at 4 °C with primary antibodies against DJ-1 (No. D29E5, Cell Signaling Technology, Beverly, CA, USA), Nrf2 (No. D1Z9C, Cell Signaling Technology, Beverly, CA, USA), Sirt1 (No. ab214185, Abcam, Cambridge, MA, USA), NLRP3 (No. ab263899, Abcam, Cambridge, MA, USA), NF-κB (No. ab32360, Abcam, Cambridge, MA, USA), Caspase-1, IL-18, and IL-1β (No. 342947, No. 516737, and No. 516288, ZEN-BIOSCIENCE, Chengdu, China) on an orbital shaker. The membranes were then washed three times with TBST and incubated with an HRP-conjugated anti-rabbit IgG secondary antibody (No. 511203, ZEN-BIOSCIENCE, Chengdu, China) for 1 h at room temperature. Protein expressions were detected using an ECL kit (No. SQ201, Epizyme, Shanghai, China) with an Immunoblotting Automatic Exposure Instrument (General Electric, Boston, MA, USA).

### 4.12. Statistical Analysis

All data are expressed as mean ± standard deviation (±s). GraphPad Prism 8.0 (GraphPad Software Inc., San Diego, CA, USA) was used for data analysis and visualization, based on three independent experiments. A one-way analysis of variance (ANOVA) was performed to assess inter-group significance. Differences were considered significant if the probability of the difference occurring by chance was less than 5% (*p* < 0.05).

## 5. Conclusions

In conclusion, our study demonstrates that FA significantly alleviates radiation-induced oxidative and inflammatory damage in intestinal tissue. This protective effect is achieved through the upregulation of the DJ-1-Nrf2 pathway and the inhibition of NLRP3 inflammasome activation, resulting in the reduced production of pro-inflammatory cytokines IL-18 and IL-1β. These findings suggest that FA holds potential as a therapeutic agent for mitigating radiation-induced intestinal injury. Looking ahead, future research should explore the development of formulations containing ferulic acid for clinical use, particularly for patients undergoing radiotherapy. Such investigations could focus on optimizing dosages and delivery mechanisms, and assessing the long-term safety and efficacy of FA in a clinical setting. Additionally, studies could explore the potential synergistic effects of FA with other therapeutic agents to enhance protective outcomes against radiation-induced damage.

## Figures and Tables

**Table 1:** Primers Sequence for qRT-PCR.

Gene	Sense (5′-3′)	Sense (5′-3′)
Sirt1	CAATCAGCTGTTGGTCAAGACT	GACAATGCAAGCTCTACCACAG
NF-κB	ATGTGGAGATCATTGAGCAGC	CCTGGTCCTGTGTAGCCATT
NLRP3	AAGGGCCATGGACTATTTCC	GACTCCACCCGATGACAGTT
Caspase-1	TCCAATAATGCAAGTCAAGCC	GCTGTACCCCAGATTTTGTAGCA
IL-18	GACCTTCCAGATCGCTTCCTC	GATGCAATTGTCTTCTACTGGTTC
IL-1β	CACGATGCACCTGTACGATCA	GTTGCTCCATATCCTGTCCCT
β-actin	ACCCAGATCATGTTTGAGAC	GCATACAGGGACAACACAG

## Data Availability

Data will be made available on request.
